# Development of a clinical prediction tool for extubation failure in pediatric cardiac intensive care unit

**DOI:** 10.3389/fped.2024.1346198

**Published:** 2024-03-05

**Authors:** Kwannapas Saengsin, Rekwan Sittiwangkul, Thirasak Borisuthipandit, Pakpoom Wongyikul, Krittai Tanasombatkul, Thanaporn Phanacharoensawad, Guanoon Moonsawat, Konlawij Trongtrakul, Phichayut Phinyo

**Affiliations:** ^1^ Division of Cardiology, Department of Pediatrics, Faculty of Medicine, Chiang Mai University, Chiang Mai, Thailand; ^2^ Center for Clinical Epidemiology and Clinical Statistics, Faculty of Medicine, Chiang Mai University, Chiang Mai, Thailand; ^3^ Division of Pulmonology and Critical Care, Department of Pediatrics, Faculty of Medicine, Chiang Mai University, Chiang Mai, Thailand; ^4^ Department of Family Medicine, Faculty of Medicine, Chiang Mai University, Chiang Mai, Thailand; ^5^ Faculty of Medicine, Chiang Mai University, Chiang Mai, Thailand; ^6^ Division of Pulmonary, Critical Care Medicine, and Allergy, Department of Internal Medicine, Faculty of Medicine, Chiang Mai University, Chiang Mai, Thailand

**Keywords:** extubation failure, pediatric cardiac patients, congenital heart disease, acquired heart disease, pediatric cardiac intensive care unit, prediction score for extubation failure

## Abstract

**Introduction/objective:**

Extubation failure in pediatric patients with congenital or acquired heart diseases increases morbidity and mortality. This study aimed to develop a clinical risk score for predicting extubation failure to guide proper clinical decision-making and management.

**Methods:**

We conducted a retrospective study. This clinical prediction score was developed using data from the Pediatric Cardiac Intensive Care Unit (PCICU) of the Faculty of Medicine, Chiang Mai University, Thailand, from July 2016 to May 2022. Extubation failure was defined as the requirement for re-intubation within 48 h after extubation. Multivariable logistic regression was used for modeling. The score was evaluated in terms of discrimination and calibration.

**Results:**

A total of 352 extubation events from 270 patients were documented. Among these, 40 events (11.36%) were extubation failure. Factors associated with extubation failure included history of pneumonia (OR: 4.14, 95% CI: 1.83–9.37, *p* = 0.001), history of re-intubation (OR: 5.99, 95% CI: 2.12–16.98, *p* = 0.001), and high saturation in physiologic cyanosis (OR: 5.94, 95% CI: 1.87–18.84, *p* = 0.003). These three factors were utilized to develop the risk score. The score showed acceptable discrimination with an area under the curve (AUC) of 0.77 (95% CI: 0.69–0.86), and good calibration.

**Conclusion:**

The derived Pediatric CMU Extubation Failure Prediction Score (*Ped-CMU ExFPS*) could satisfactorily predict extubation failure in pediatric cardiac patients. Employing this score could promote proper personalized care. We suggest conducting further external validation studies before considering implementation in practice.

## Introduction

Extubation failure substantially potentiates morbidity and mortality in pediatric intensive care units (PICUs). The prevalence of extubation failure ranges from 5% to 35% (1–3). Various studies have demonstrated risk factors associated with extubation failure in pediatric patients, including genetic syndrome, younger age, prolonged mechanical ventilation, sedation of longer than five days, post-extubation stridor (PES), respiratory muscle weakness, uncuffed endotracheal tube use, and a set of positive end-expiratory pressure (PEEP) greater than 5 cmH_2_O (4–6).

Pediatric patients with congenital or acquired heart diseases pose challenges when considering extubation due to the lack of comprehensive understanding of these conditions (7, 8). Complex congenital heart diseases can lead to abnormal pulmonary (Qp) and systemic (Qs) blood flow distribution. Furthermore, PES, which is a risk factor after extubation, could disrupt the limited cardiopulmonary reserves and contribute to an augmentation in stress affecting the ventricular walls of pediatric cardiac patients (4). Therefore, understanding the patient's hemodynamics status relying on Qp/Qs optimization and general factors related to extubation failure is crucial for intubated pediatric cardiac patients (9–12).

The ability to predict extubation failure could offer significant benefits to clinicians and intensivists (1). It can aid in formulating more effective management plans, potentially leading to improved patient outcomes such as shorter ICU stays and reduced morbidity and mortality (2). However, most prediction models for extubation failure in pediatrics have been conducted in preterm and general pediatric populations (11–13). To the best of our knowledge, there hasn't been a clinical prediction score developed specifically for pediatric cardiac patients. Thus, our goal was to develop a clinical score to assess extubation failure risks in this group. Additionally, we intended to evaluate the potential added value of PES, a predictive factor that may be worthwhile to monitor during the 48 h following extubation (8, 13).

## Materials and methods

### Study design and population

A prognostic prediction research was conducted using a retrospective study design, from July 2016 to May 2022. The data related to every extubation event of patients admitted to the Pediatric Cardiac Critical Care Unit (PCICU) of Chiang Mai University Hospital were gathered and collected. The Faculty of Medicine, Chiang Mai University's institutional review board approved this study (PED-2565-08935). The ethics institutional review board waived the requirement of written informed consent because this research was conducted retrospectively and involved no more than minimal risk to subjects. Patient data were pseudonymized to prevent direct identification and maintain patients' confidentiality. We adhered to the Statistical Analyses and Methods in Published Literature (SAMPL) and Transparent Reporting of a multivariable prediction model for Individual Prognosis Or Diagnosis (TRIPOD) guidelines for study reporting (14, 15).

### Inclusion and exclusion criteria

The inclusion criteria included pediatric patients with congenital or acquired heart diseases older than one month and less than 18 years who received MV support. Patients who were on extra corporeal membrane oxygenation (ECMO) in the PCICU were also included. We excluded tracheostomized patients, patients who passed away or withdrew life support while receiving mechanical ventilation, patients discharged with mechanical ventilation, and those who had undergone unplanned extubation.

### Weaning protocol

In our clinical practice for weaning MV, weaning begins after the patient's hemodynamic status was stable. The vital signs were normal for age. The SpO_2_ > 95% in physiologic acyanosis and SpO_2_ > 70% in physiologic cyanosis. We utilized a low-level pressure support ventilator (PSV) to achieve a minimum tidal volume (TV) of 6–8 ml/kg with continuous positive airway pressure (CPAP) of 4–5 cmH_2_O for at least 30 min. Additionally, the level of pressure support was adjusted to overcome endotracheal tube (ETT) resistance as follows: ETT size 3.0–3.5, PS = 10 cmH_2_O; ETT size 4.0–4.5, PS of 8–9 cmH_2_O; ETT size greater than 5.0, PS of 6–7 cmH_2_O. We titrated a minimum FiO_2_ below 40% to achieve optimal oxygen saturation according to physiologic conditions.

In our practice, after a successful 30-min weaning period, doctors decide to extubate the ETT if the patient is in good consciousness. The respiratory rate remains within the normal range for their age group and shows no signs of respiratory distress, such as sternocleidomastoid muscle contraction or the presence of intercostal or subcostal retractions. The respiratory rate for age are as follows: 20–60/min for 1–6 months, 15–45/min for 6 months to 2 years, 15–40/min for 2–5 years, and 10–35/min for older than 5 years (16).

The patient's heart rate should not deviate more than 20% from baseline. Additionally, we observe cough strength during suction in those patients who cannot communicate. For those patients using a non-cuffed ETT, we apply a brief period of pressure at 20 cmH_2_O and observe for cuff leak using auscultation over the trachea. For patients receiving a cuffed ETT, we deflate the cuff, ventilate the patient, auscultate over the patient's trachea, and listen for air turbulence. If the cough is adequate and the leak test is positive, then we proceed to extubate the ETT.

### Data collection

We collected the following patient data: demographics, baseline clinical characteristics, history of cardiac surgery, history of intubation, and treatment during intubation. Vital signs and clinical conditions were collected within 2 h before ETT extubation. The laboratory data within the prior 72 h were reviewed. The data closest to the time of extubation was chosen. We also reviewed MV parameters during weaning, including the ventilator mode, peak inspiratory pressure (PIP), PEEP, ETT size, and ETT type. All data were retrieved from electronic medical records and registered in RedCap (Vanderbilt University, Nashville, Tennessee).

### Candidate predictors and definitions of predictors

We prespecified ten potential predictors for developing a score using prior knowledge, clinical expertise, and a thorough clinical literature review: body mass index (BMI) (17); history of reintubation was defined as the patient experienced re-intubation during PCICU admission (18); history of pneumonia was defined as the presence of fever, productive sputum, identification of lung infiltration in chest x-ray (CXR), and/or positive findings in sputum culture before extubation which included community-acquired pneumonia (CAP), hospital-acquired pneumonia (HAP), and ventilator-associated pneumonia (VAP) (19, 20); respiratory rate (1); physiologic cyanosis was defined as the peripheral oxygen saturation (SpO_2_) of the patient during intubation at less than 95% e.g., single ventricle physiology, Tetralogy of Fallot (TOF) status post (s/p) modified right Blalock–Thomas–Taussig shunt; physiologic acyanosis was defined as the SpO_2_ of the patient during intubation at ≥95% e.g., atrial septal defect and TOF s/p total correction; palliative surgery was defined as the operative surgery as follows: systemic to pulmonary artery (PA) shunt, PA banding, bidirectional cavopulmonary anastomosis, Norwood operation, Yasui operation, or Nikaidoh operation (18); genetic syndrome (1, 2); duration ETT of longer than seven days (8, 13, 21, 22); uncuff ETT (4); intravenous fentanyl administration or intravenous midazolam administration of longer than five days (8, 21). All potential predictors are determined pre-extubation. However, PES is observed post-extubation and is widely recognized in the literature as a potential risk factor (8, 13, 22). As a result, we later incorporated it into our model and assessed the added value of observing PES post-extubation. PES is defined as the diagnosis of PES or the occurrence of clinical inspiratory stridor within 24 h after extubation, as determined by a review of the medical records.

### Study endpoint

The study endpoint was extubation failure which was defined as a re-intubation within 48 h (7) as noted from the medical record. In our center, the respiratory failure needed for reintubation was based on clinical presentation by increased respiration rate, increased respiratory muscle use, desaturation from baseline (physiologic acyanosis-SpO_2_ < 95%, physiologic cyanosis- SpO_2_ < 70%), and no improvement in clinical status after applying non-invasive ventilation. The respiratory failure was determined by the clinical judgment of individual clinicians working at that time.

### Data analysis

In this study, each extubation attempt served as an individual unit of analysis, allowing for the possibility of one patient being represented in multiple occurrences in the dataset. Categorical variables were reported as frequencies and percentages, while continuous variables were expressed as either mean with standard deviation (SD) or median with interquartile range (IQR), depending on their distribution. Statistical comparison for categorical variables utilized the Pearson-Chi-squared test. For continuous variables, either the student *t*-test or Mann–Whitney *U*-test was employed, depending on the distribution. A *p*-value below 0.05 indicated statistical significance. Statistical analysis was performed using Stata 17 (StataCorp, College Station, Texas, USA).

### Study size estimation

To estimate the minimum sample size required for developing a multivariable prediction model for binary outcomes, the expected incidence of extubation failure from previous study was 35% (3, 23). We designated 10 as the number of candidate predictors and set the shrinkage factor at 0.9, anticipating a model C-statistic of 0.8. The minimum sample size required for new model development, based on user inputs, was calculated as 350 with 123 events (assuming an outcome prevalence of 35%) and an event per predictor of 12.25.

### Prediction score development

We examined all variables for collinearity and selectively included pre-selected predictors in the model. To handle missing data, we followed James R. Carpenter's framework (24). We evaluated the appropriateness of complete case analysis to determine if it is likely to produce biased results. If the evaluation indicates that complete case analysis is unlikely to introduce bias, it will be applied. We performed univariable logistic regression analyses to evaluate the relationship between each variable and the occurrence of extubation failure. Finally, pre-selected predictors were integrated into the multivariable logistic regression model. We initially included all predictors in the full model and conducted backward elimination, removing those with a *p*-value greater than 0.05 from the model. After model reduction, we calculated the score for each predictor by dividing the regression coefficients of all final predictors with the smallest predictor's coefficient and rounding up (25). The total score for the Pediatric CMU Extubation Failure Prediction Score (*Ped-CMU ExFPS*) was obtained by summing these rounded coefficients. We provided a logistic equation to be used for calculating the probability of extubation failure and model evaluation in further studies. Two logistic equations were provided: (1) the equation for the final model before score transformation, and (2) the equation of the model where transformed scores were used as a sole predictor. The first equation can be used to predict the probability based on the crude logit coefficient. The second equation can be used to predict the probability based on the transformed score. The predicted probability of extubation failure can be calculated using the following formula, where *e* is the base of the natural logarithm, and *α* is the model intercept, and *β* is the beta coefficient for each model predictor:

$$\mathrm{Predicted}\;\mathrm{probability}\;\mathrm{of}\;\mathrm{extubation}\;\mathrm{failure}=\frac{e^{\left(\alpha +\beta x\right)}}{1+e^{\left(\alpha +\beta x\right)}}.$$

### Prediction score performance

We assessed the score predictive performance by using the model's discrimination and calibration. The discriminative ability of the score was based on the area under the curve (AUC). To assess score calibration, we graphed a calibration plot and performed a Hosmer–Lemeshow goodness-of-fit statistical testing.

### Internal validation

For internal validation, we performed with a bootstrap resampling with 500 replicates to assess the optimism of the model.

### Additional analyses

When incorporated PES into the *Ped-CMU ExFPS* model, the added value of the predictor was tested using the comparison of the areas under two correlated receiver operating characteristic curves (26). We also conducted a *post hoc* subgroup analysis to examine the differences in model discrimination in infant and non-infant groups.

## Results

### Study population

**Figure 1:**
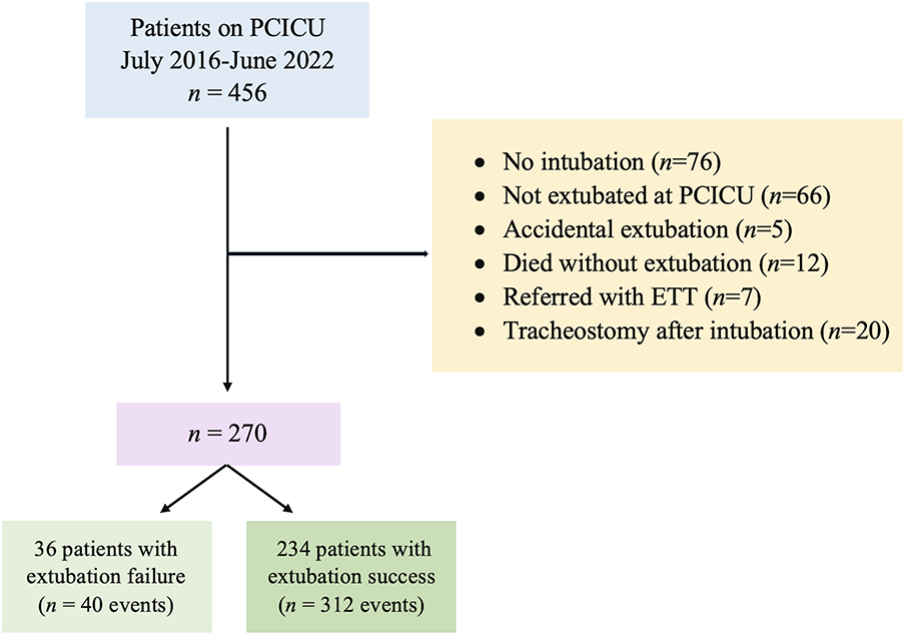
Flowchart of the study cohort [adapted from (18)]. ETT, endotracheal tube; PCICU, pediatric cardiac intensive care unit.

Our cohort had 352 extubations involving 270 pediatric cardiac patients (Figure 1). In this study, only one patient who required ECMO was included. There were 40 (11.36%) extubation failures. Notably, no patients required re-intubation for a procedure. Of the 40 events of extubation failure, 33 events were the first time of extubation failure. Two patients experienced extubation failure twice. The patients were diagnosed with D-transposition of the great artery with ventricular septal defect and TOF. One patient who was diagnosed with pulmonary atresia intact ventricular septum experienced extubation failure three times. Demographic data are provided in Table 1, revealing a significantly lower BMI in the extubation failure group compared to the extubation success group (*p* = 0.004). Additionally, the extubation failure group had a higher prevalence of palliative surgery. Genetic syndrome was identified in 30.40% (107/352). The three most common cardiac diagnoses among these patients were ventricular septal defect (21.88%), functional single ventricle (17.61%), and TOF (15.06%).

**Table 1:** Baseline demographic and clinical characteristics of patients.

Variables	All extubation (*n* = 352)	Failure (*n* = 40)	Success (*n* = 312)	*p*-Value
Age (months)^a^	8.96 (2.85,21.84)	6.51 (1.95,18.45)	9.44 (2.92,22.37)	0.139
Infant (30 day to 1 year) (*n*,%)	205 (58.24)	27 (67.50)	178 (57.05)	0.236
Child (1–18 year) (*n*,%)	147 (41.76)	13 (32.50)	134 (42.95)	
male, *n* (%)	182 (51.70)	22 (55)	160 (51.28)	0.738
Weight (kg)^a^	5.71 (3.7,9.0)	4.7 (3.25,7.9)	5.8 (3.78,9.3)	0.094
Height (cm)	64 (54.2,78)	60.5 (54,75)	64 (54.75,78.5)	0.338
Body mass index (kg/m^2^)	14.14 (3.34)	12.69 (2.79)	14.32 (3.36)	0.004
Genetic syndrome, *n* (%)	107 (30.40)	12 (30)	95 (30.45)	0.557
Down syndrome	47 (43.95)	5 (41.67)	42 (44.21)	0.721
Heterotaxy syndrome	25 (23.36)	3 (25.00)	22 (23.16)	
22q 11 syndrome	13 (12.15)	1 (8.33)	12 (12.63)	
VACTERL association	3 (2.80)	1 (8.33)	2 (2.11)	
Others	19 (17.76)	2 (16.67)	17 (17.89)	
Primary cardiac diagnosis, *n* (%)				
Ventricular septal defect	77 (21.88)	7 (17.50)	70 (22.44)	0.886
Functionally single ventricle	62 (17.61)	11 (27.50)	51 (16.35)	
Tetralogy of Fallot	53 (15.06)	6 (15.00)	47 (15.06)	
D-transposition of great artery	24 (6.82)	2 (5.00)	22 (7.05)	
Patent ductus arteriosus	19 (5.40)	3 (7.50)	16 (5.13)	
Double outlet right ventricle	17 (4.38)	1 (2.50)	16 (5.13)	
Atrioventricular septal defect	14 (3.39)	1 (2.50)	13 (4.17)	
Coarctation of aorta/interrupted aortic arch	13 (3.69)	1 (2.50)	12 (3.85)	
Others	73 (20.74)	8 (20.00)	65 (20.83)	
Native anatomy cyanosis	175 (49.72)	22 (55.00)	153 (49.04)	0.506
Post-operative surgery, *n* (%)	208 (59.09)	23 (57.50)	185 (59.29)	0.865
Palliative surgery	79 (37.98)	16 (69.57)	63 (34.05)	0.001
Total correction	129 (62.02)	7 (30.43)	122 (65.95)	
Post-intervention, *n* (%)	4 (1.14)	0 (0)	4 (1.28)	1.000

IQR, inter-quartile range; SD, standard deviation; VACTERL, vertebral defects, and atresia, cardiac defects, trachea-esophageal fistula, renal anomalies, and limb abnormalities.

Continuous data were expressed as mean (SD).

^a^ Otherwise, Denoted median (IQR).

### Factors associated with extubation failure

We conducted univariable logistic regression analyses to identify predictors associated with extubation failure in Table 2 and Supplementary Table S1. Factors associated with an increased risk of extubation failure included low BMI, post-operative palliative surgery, history of reintubation, history of pneumonia, high respiratory rate, physiologic cyanosis, and PES. We further evaluated and found that 178 out of 352 events involved patients who received steroids before extubation. One hundred and fifty-five events were in the extubation success group (49.69%), and 23 events were in the extubation failure group (57.50%). There was no statistical difference between the two groups of patients (*p* = 0.223).

**Table 2:** Potential predictors of extubation failure based on univariable logistic regression analysis.

Variable	OR	95% CI		*p*-Value	ROC	95% CI	
		Lower	Upper			Lower	Upper
Medical conditions before extubation							
Age, months	0.99	0.98	1.00	0.376	0.57	0.47	0.70
Infant	1.56	0.78	3.14	0.210	0.55	0.47	0.63
Male	1.61	0.59	2.24	0.658	0.52	0.44	0.60
Weight, kg	0.93	0.85	1.01	0.087	0.58	0.49	0.68
Height, cm	0.99	0.97	1.00	0.325	0.55	0.45	0.64
BMI, kg/m^2^	0.80	0.69	0.92	0.002	0.67	0.58	0.76
Genetic syndrome	0.97	0.47	2.00	0.954	0.50	0.43	0.58
Cyanotic heart disease	1.27	0.65	2.46	0.478	0.53	0.45	0.61
Post-operative surgery	0.85	0.44	1.61	0.611	0.52	0.44	0.60
Post-operative palliative surgery	4.43	1.73	11.32	0.002	0.68	0.58	0.78
Post-operative open-heart surgery	0.25	0.09	0.65	0.005	0.62	0.56	0.68
History of reintubation in admission	7.51	3.16	17.86	<0.001	0.61	0.54	0.68
Intubation >7 days	1.76	0.91	3.41	0.094	0.57	0.49	0.65
VIS before extubation 48 h	1.01	0.95	1.07	0.816	0.51	0.43	0.58
Continuous Sedation before extubation >48 h	1.08	0.54	2.19	0.824	0.51	0.43	0.59
Duration of muscle relaxant, day	0.93	0.76	1.15	0.518	0.52	0.47	0.57
Duration of Fentanyl >5 days	1.10	0.50	2.43	0.808	0.51	0.44	0.57
Duration of Midazolam >5 days	0.83	0.28	2.45	0.730	0.51	0.46	0.56
CXR atelectasis before extubation	2.44	0.64	9.30	0.188	0.52	0.48	0.56
History of pneumonia before extubation	3.99	1.93	8.29	<0.001	0.66	0.58	0.74
Pulmonary hypertension	0.47	0.21	1.04	0.064	0.57	0.51	0.64
Fluid balance, ml/kg	1.00	0.99	1.02	0.240	0.55	0.46	0.64
Vital signs and clinical condition before extubation							
Respiratory rate, beats/min	1.05	1.02	1.09	0.003	0.62	0.53	0.72
Heart rate, beats/min	1.01	0.99	1.03	0.120	0.59	0.51	0.67
Body temperature, c	0.99	0.85	1.14	0.838	0.57	0.48	0.66
Systolic blood pressure, mmHg	0.98	0.96	1.01	0.133	0.56	0.45	0.67
Diastolic blood pressure, mmHg	0.99	0.96	1.01	0.375	0.55	0.45	0.66
Mean arterial blood pressure, mmHg	0.98	0.96	1.01	0.235	0.56	0.45	0.67
Physiologic saturation							
Cyanosis (SpO_2 _ _ _85)	Ref						
Acyanosis (SpO_2 _> 95)	0.84	0.32	2.17	0.714			
Cyanosis (SpO_2_ > 85)	5.34	2.47	11.69	<0.001	0.63	0.54	0.72
Ventilator and testing before extubation							
Mode of ventilator					0.58	0.51	0.66
PS	Ref						
SIMV with PS	0.74	0.37	1.47	0.387			
PC	0.15	0.02	1.14	0.067			
ETT size	0.66	0.38	1.14	0.142	0.57	0.48	0.67
ET tube cuff	0.67	0.27	1.68	0.398	0.53	0.47	0.59
Peak pressure, cmH_2_O	1.02	0.89	1.18	0.757	0.51	0.41	0.61
PEEP, cmH_2_O	1.20	0.83	1.74	0.327	0.56	0.46	0.65
Clinical condition after extubation							
Post extubation stridor	3.67	1.86	7.19	<0.001	0.65	0.57	0.73

BMI, body mass index; CI, confidence interval; CXR, chest x-ray; ET, endotracheal tube; PEEP, positive end-expiratory pressure; PC, pressure control; PS, pressure support; ROC, a receiver operating characteristic curve; SIMV, synchronized intermittent mandatory ventilation; VIS, Vasoactive-Inotropic Score.

Missing data Intake output (balanced) *n = *9, respiratory rate *n = *1, heart rate *n = *1, body temperature *n = *1, systolic blood pressure *n = *1, mean arterial blood *n = *1, mode of ventilator blood *n = *4, ET tube cuff *n = *2, peak pressure *n = *6, PEEP *n = *5.

### Prediction score for extubation failure for pediatric cardiac patients

**Figure 2:**
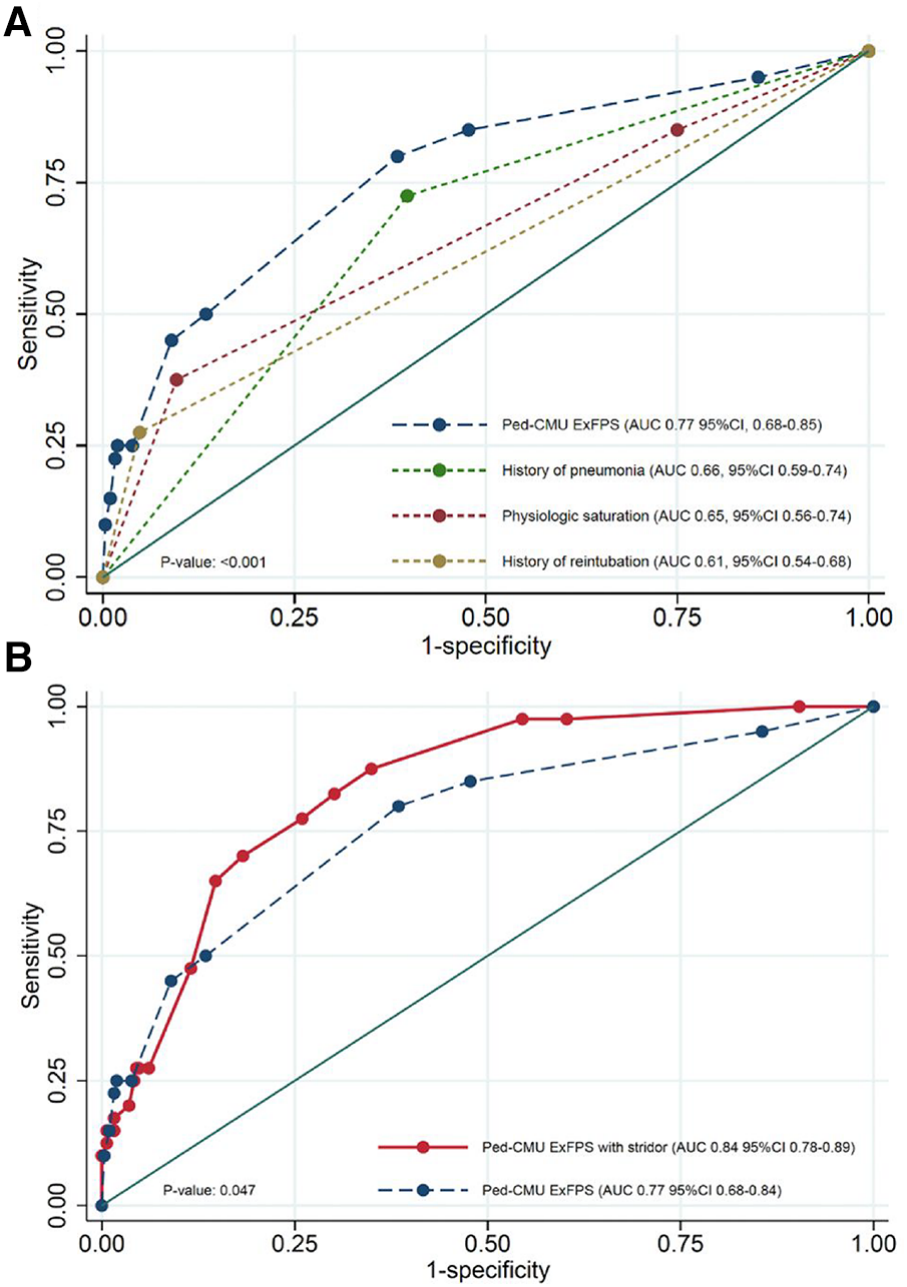
(**A**) AUC curves for *Ped-CMU ExFPS* from derivation cohort was higher than the AUCs of the individual parameters; (**B**) comparison between AUC of *Ped-CMU ExFPS* (blue line) and *Ped-CMU ExFPS* with PES (red line). AUC: the receiver operating characteristic; *Ped-CMU ExFPS*, pediatric CMU extubation failure predictive score; PES, post-extubation stridor.

The full multivariable logistic regression model included ten pre-selected candidate predictors. The missing data in our study was less than 5% and did not affect the extubation failure outcome, therefore, the complete case analysis was applied. Three significant predictors included the history of reintubation, the history of pneumonia, and a physiologic saturation with an AUC of 0.82 (95% CI: 0.76, 0.88), as shown in Table 3*.* The *Ped-CMU ExFPS* score ranges from zero to twenty points. The highest score was for reintubation (10 points), followed by physiologic saturation in cyanosis patients with SpO2 > 85 (6 points), pneumonia (4 points), and by physiologic saturation: acyanosis (1 point). After using all three pre-extubation predictors to develop the *Ped-CMU ExFPS* model, we found a good discriminative ability of this model with an AUC of 0.77 (95% CI: 0.69,0.86) (Figure 2). The *Ped-CMU ExFPS* showed good calibration when comparing predicted risk to the observed outcomes (Supplementary Figure S1). Additionally, the Hosmer–Lemeshow goodness of fit statistic for this score was not insignificant (*p* = 0.669). The equation for the final model before score transformation is as follows:

$$\begin{array}{r} \mathrm{Predicted}\;\mathrm{probability}\;\mathrm{of}\;\mathrm{extubation}\;\mathrm{failure}\\ =\frac{e^{(-3.576+(1.912\times \mathrm{Retube}\;)+(1.417\times \mathrm{Pneu})+(0.217\times \mathrm{Physio}\_\mathrm{acy})+(1.584\times \mathrm{Physio}\_\mathrm{cya}))\;}}{1+e^{(-3.576+(1.912\times \mathrm{Retube})+(1.417\times \mathrm{Pneu})+(0.217\times \mathrm{Physio}\_\mathrm{acy})+(1.584\times \mathrm{Physio}\_\mathrm{cya}))\;}} \end{array}$$

**Table 3:** Prediction score for extubation failure of pediatric cardiac patients.

Variable	Full model (*n* = 349)				Reduced model (*n* = 352)				*β*	*p*-Value	Assign score
	OR	95% CI		*p*-Value	OR	95% CI		*p*-Value			
		Lower	Upper			Lower	Upper				
Reintubation	5.31	1.71	16.49	0.004	6.76	2.54	18.01	<0.001	1.91	<0.001	10
Pneumonia	4.44	1.91	10.31	0.001	4.12	1.87	9.06	<0.001	1.42	<0.001	4
Physiologic saturation											
Cyanosis (SpO_2_≤85)	Ref				Ref				Ref		
Acyanosis (SpO_2_ > 95)	1.26	0.45	3.47	0.659	1.24	0.46	3.36	0.668	0.22	0.668	1
Cyanosis (SpO_2_ > 85)	4.53	1.44	14.29	0.010	4.87	1.61	14.71	0.005	1.58	0.005	6
BMI	0.87	0.75	1.00	0.063							
Respiratory rate	1.03	0.99	1.07	0.134							
Duration of intubation >7 days	0.88	0.36	2.11	0.767							
Duration of Fentanyl > 5 days	1.03	0.37	3.25	0.958							
Duration of Midazolam > 5 days	1.38	0.33	5.85	0.662							
Genetic	1.69	0.78	3.91	0.221							
ET tube with cuff	0.64	0.21	1.97	0.442							

CI, confidence interval; ET, endotracheal tube.

Intake output respiratory rate *n = *1, ET tube with cuff *n = *2.

The details on the four predictors within the equation are as follows:

• Retube: history of reintubation in admission [Yes = 1 No = 0]
• Pneu: history of pneumonia before extubation [Yes = 1 No = 0]
• Physio_acy: physiologic acyanosis [Yes = 1 No = 0]
• Physio_cya: physiologic cyanosis with oxygen saturation higher than 85 [Yes = 1 No = 0]

The equation for the score model is as follows:

$$\mathrm{Predicted}\;\mathrm{probability}\;\mathrm{of}\;\mathrm{extubation}\;\mathrm{failure}\;=\frac{e^{\left(-3.286+0.231\times Ped-CMU\;ExFPS\;score\;\right)}}{1+e^{\left(-3.286+0.231\times Ped-CMU\;ExFPS\;score\;\;\right)}}$$

### Internal validation

Internal validation of the *Ped-CMU ExFPS* with a bootstrapping procedure using 500 replicates showed an apparent AUC of 0.80 (range 0.72–0.88) and a test AUC of 0.76 (range 0.68–0.83). The optimism of AUC was 0.05 (range 0.04–0.05) (Supplementary Figure S2).

### Additional analyses

PES had the potential added value for predicting extubation failure. The discriminative ability was improved after incorporating PES into the model by increase in AUC from 0.77 (95% CI: 0.69, 0.86) to 0.84 (95% CI: 0.76, 0.89), *p* = 0.047 (Figure 2B). The performance of the *PED-CMU ExFPS* in the non-infant group was AUC of 0.65, (95% CI: 0.47–0.82), whereas the performance of the *PED-CMU ExFPS* in the infant group was AUC of 0.82, (95% CI 0.74–0.91); the difference was not statistically significant (*p*-value = 0.071).

### Clinical application for the Ped-CMU ExFPS score

**Figure 3:**
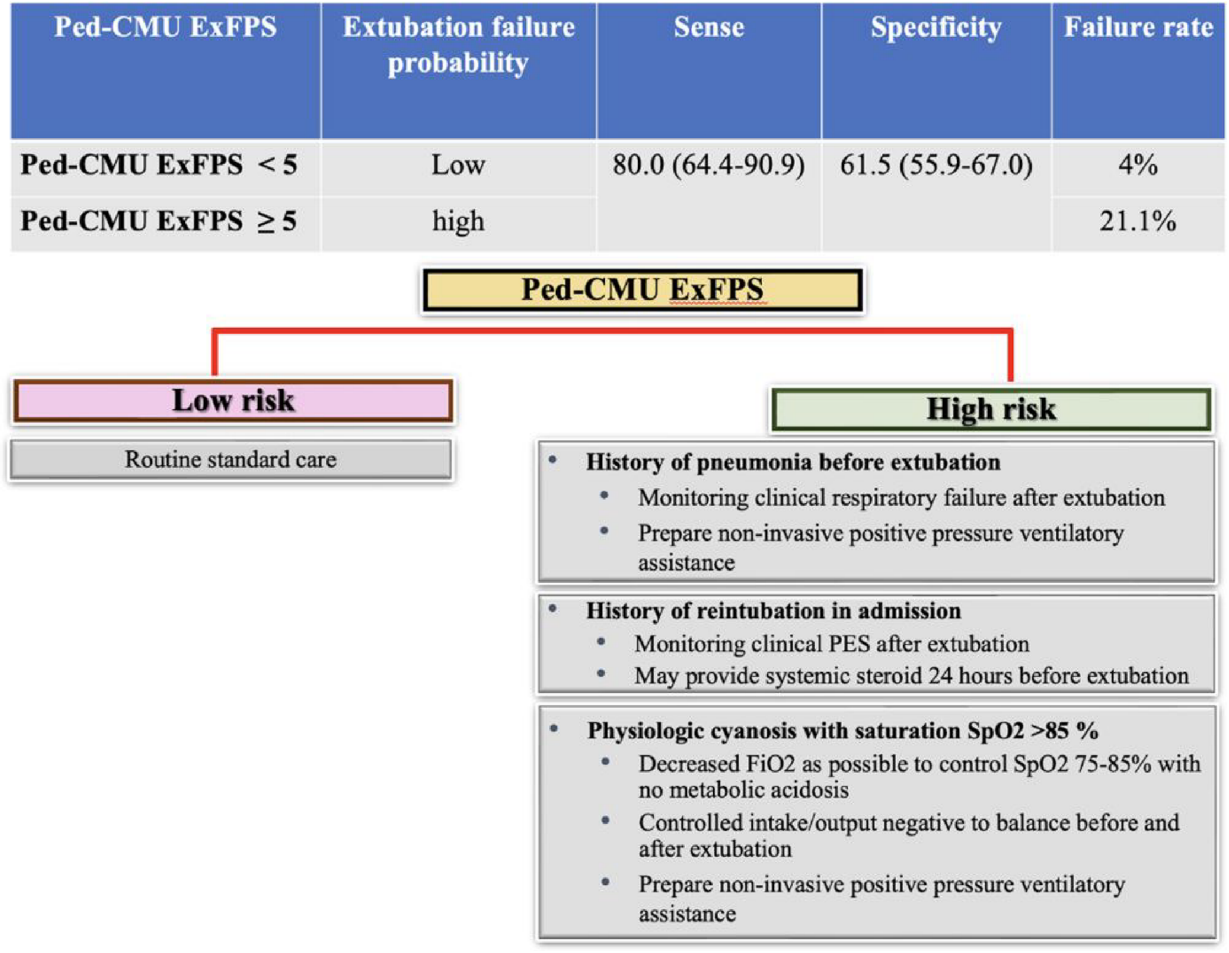
An infographic of the predictive risk of extubation failure outcomes and guidance for proper management.

For clinical applicability, the derived models are presented as an easy-to-use web application (https://ped-cmu-exfps.web.app) (Supplementary Figures S3 and S4). *Ped-CMU ExFPS* is based on individual inputs. The application would estimate the predicted probability of extubation failure within 48 h after extubation in pediatric cardiac patients. We categorized the scores into two clinical risk categories: low- and high-risk. *Ped-CMU ExFPS* score below five points suggests a low probability of extubation failure, with a 4% risk identified. On the other hand, when the *Ped-CMU ExFPS* score reaches five or higher, the probability of extubation failure rises substantially, reaching 21.1%. The sensitivity and specificity levels were 80% (95% CI: 64.4–90.9) and 61.5% (95% CI: 55.9–67.0), respectively (Figure 3, Supplementary Figure S3).

## Discussion

This study developed the novel prediction model “*Ped-CMU ExFPS*,” a web-based application for predicting extubation failure in pediatric cardiac patients. Our final model comprised three routinely available predictors: history of reintubation in admission, history of pneumonia before extubation, and physiologic saturation. *Ped-CMU ExFPS* showed good discrimination performance and was well-calibrated. *Ped-CMU ExFPS* aims to predict the probability of extubation failure before extubation and provide clinicians with suitable suggestive management strategies for individual risk factors.

This study showed that extubation failure in PCICU occurred in 11.36% of extubation events. Our study aligns with previous studies, indicating that extubation failure in the PICU varies between 5% and 35%, influenced by the specific underlying disease and illness severity (1, 2). Genetic syndrome was found in 30.4% of all extubation events (107/353). Down syndrome was most common. Salgado et al. reported extubation failure after cardiac surgery in children with down syndrome. They were younger age, presence of aortic coarctation, higher cardiomegaly and hypotonia (27).

Our *Ped-CMU ExFPS* provided the predictive risk of extubation failure. One of the risk factors of extubation failure in PCICU is the history of pneumonia in admission. Pneumonia-induced lung inflammation and fluid collection in alveoli result in a decreased ability for gas exchange and an elevation in the respiratory effort needed (28). Abnormal lung function may be one of the risk factors for extubation failure (20). In our center, we suggest carefully observing patients for clinical indicators of respiratory distress following extubation. If respiratory distress occurs, readiness to employ non-invasive ventilatory support is essential. Non-invasive ventilatory support has been considered valuable in preventing reintubation by improving mucociliary clearance, helping lung re-expansion, and improving pulmonary ventilation (29).

History of reintubation in admission possesses the capacity to injure the mucosal layer, resulting in tearing, injury to the airway, and swelling, potentially leading to PES. The process of re-intubation can extend the overall duration of intubation within the PCICU. Findings from previous studies have indicated that prolonged intubation contributes to the risk of extubation failure (2, 30). The consideration of steroid prophylaxis before extubation could be worth exploring. Furthermore, thorough monitoring and preparedness for suitable PES management following extubation may be advisable (31).

Pediatric cardiac patients with native complex anatomy or post-operative surgery may have abnormal distribution of pulmonary blood flow (Qp) and systemic blood flow (Qs). An increase in Qp can result in pulmonary overcirculation and decreased systemic circulation. This could potentially contribute to extubation failure due to increased pulmonary blood flow (9–12). Balancing Qp/Qs before extubation may decrease the risk of extubation failure. Arterial oxygen saturation (SaO_2_) has become the target for balancing pulmonary blood flow and systemic circulation. SaO_2_ of 75%–85% is believed to reflect a balanced circulation with Qp/Qs of 1 (9). Given the robust correlation between SaO_2_ and SpO_2_, SpO_2_ provides a convenient and non-invasive technique to approximate SaO_2_ (32). Our findings align with the previous literature. We found that in a group with physiologic cyanosis, high SpO_2_ > 85 was a risk factor for extubation failure due to high pulmonary blood flow. We suggest maintaining SpO_2_ within the 75%–85% range to achieve a balanced pulmonary blood flow and optimize systemic oxygen delivery. Oxygen is a potent pulmonary vasodilator that could increase pulmonary blood flow (33). This can be achieved by adjusting FiO_2_ to its lowest possible level for the optimum target SpO_2_ without causing metabolic acidosis. When saturation remains elevated despite FiO_2_ adjustment, it may cause a potential increase in the risk of hemodynamic pulmonary overcirculation. Balance intake output may be needed. After extubation, patients should be closely monitored, and if they experience respiratory distress, it is crucial to be prepared to utilize non-invasive ventilatory assistance.

PES can lead to upper airway obstruction associated with mechanical ventilation under endotracheal intubation. The mechanical force or irritation from the endotracheal tube can contribute to the potentially developed laryngeal swelling (31). Patients with PES may likely have increased re-intubation incidences (13, 34, 35). A previous study reported that patients with PES encountered a re-intubation rate of 47.4%, indicating an elevation of 5.7 times the standard population average (8). In pediatric cardiac patients, the elevation in airway resistance can alter the limited cardiopulmonary reserve and increase ventricular wall stress (4). Our study showed the same result as the previous studies. PES, a post-extubation factor, contributed as an added value of predictive factors for extubation failure in pediatric cardiac patients. This finding may guide physicians to provide systemic corticosteroid administration before extubation in patients with a risk of PES. Furthermore, preparing therapeutic strategies to reduce the risk of reintubation from PES is crucial. These interventions could involve adrenaline or steroid nebulization after extubation, systemic steroid administration for reducing airway inflammation, or an application of non-invasive ventilatory assistance for alleviating anatomical dead space, decreasing subglottic laryngeal inflammation, and reducing airway resistance (31, 36–38).

In this study, we employed the *Ped-CMU ExFPS* with a threshold set at 5 to categorize cases as having a low probability of extubation failure. The chosen cut-off point yielded a sensitivity of 80% and specificity of 61%. Our goal is to minimize the occurrence of false negatives and enhance the positive predictive value. The rationale behind prioritizing few false negatives lies in the potential severe consequences associated with missing cases of extubation failure, which can lead to elevated morbidity and mortality.

The field of prediction modeling for extubation failure in pediatrics is limited, with most research efforts directed toward preterm and the general pediatric population (39–41). Our study might be one of the first prediction scores to predict extubation failure in pediatric cardiac patients. We incorporated routinely available predictors and physiologic hemodynamics to customize individual guides based on the risk factors for each patient.

### Study limitations

Our study has some limitations. Firstly, as part of a retrospective study, data collection might be subject to bias and have missing data. In addition, the routine protocol for weaning and extubation during the study period might need to be sufficiently controlled as in trials or prospective studies. However, the data missingness was low. Secondly, our model can be utilized only for children older than one month, which affects the model's generalizability. Applying our model to neonatal patients is not possible, as this population was excluded from the study.

Thirdly, we did not have data on the use of pulmonary vasodilators, such as inhaled nitric oxide (iNO), sildenafil, and calcium antagonists in patients with pulmonary hypertension, which could also be associated with pulmonary blood flow and extubation failure. Fourthly, the *post hoc* subgroup analysis revealed that the discriminative ability of the model in the overall sample used was driven mainly by infant patients, whereas the performance in the non-infant group was not as high. Therefore, this might imply that our model might be more beneficial to infant patients. Nonetheless, we suggested that this finding be validated in external data with a higher proportion of non-infant patients. Finally, in our study, with an observed incidence of extubation failure at 11.4%, we obtained fewer events than expected—only 40 events instead of the anticipated 123. This limitation significantly impacts our ability to address overfitting and achieve precise estimation in the prediction model. Nonetheless, our internal validation indicated that the degree of overfitting might not be significant. As this study was conducted within a single center, the generalization or transportability could not be guaranteed. Therefore, further studies to validate our newly-developed score are needed.

## Conclusion

The *Ped-CMU ExFPS*, incorporating factors of history of pneumonia, history of reintubation, and physiologic saturation, offers a reliable and satisfactory prediction of extubation failure in pediatric cardiac patients. The utilization of this score may enhance the provision of individualized care and simplify practical risk assessment. For patients with PES, close monitoring after extubation is particularly essential.

## Data Availability

The raw data supporting the conclusions of this article will be made available by the authors, without undue reservation.
