# Supplementary material for: Development of a clinical prediction tool for extubation failure in pediatric cardiac intensive care unit
Source: Front Pediatr. 2024 Mar 5;12:1346198. doi: 10.3389/fped.2024.1346198 (PMC10948403; doi:10.3389/fped.2024.1346198)
Supplement: Supplementary file 1 [file Datasheet1.pdf]

Supplementary Material

1 Supplementary Figures and Tables

1.1 Supplementary Figures

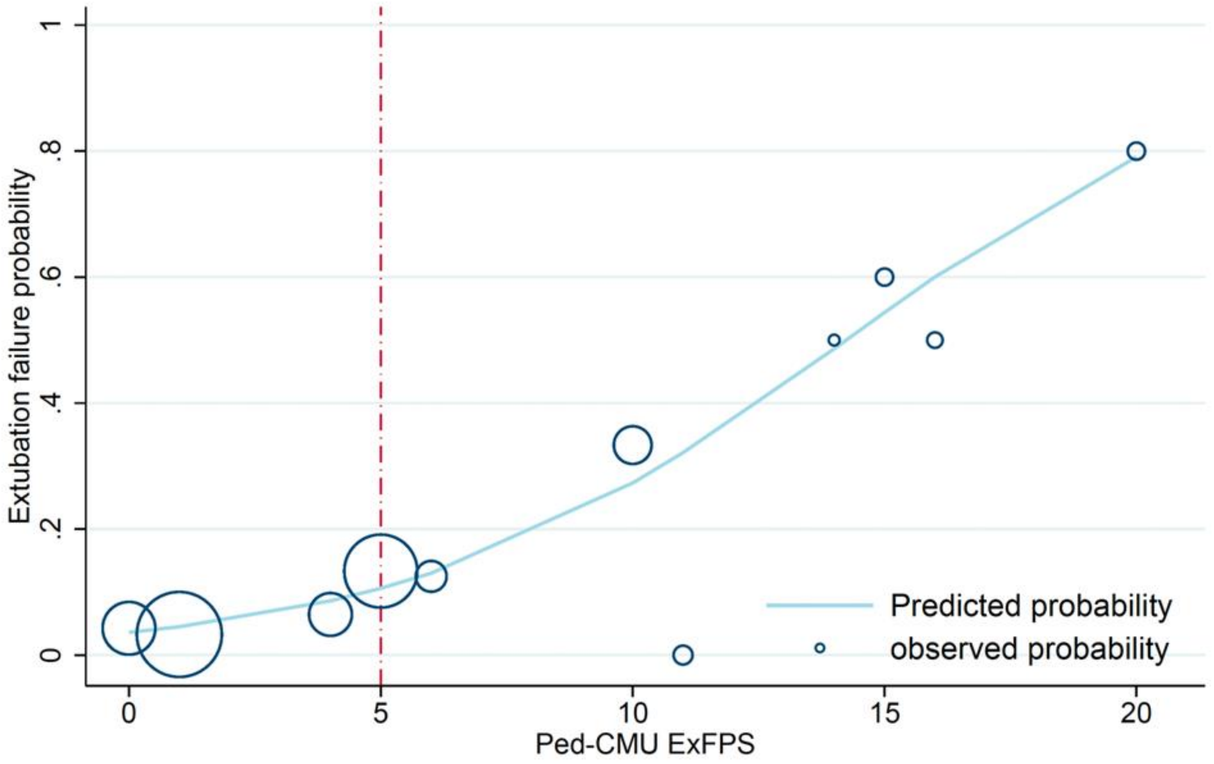

Supplementary Figure 1. Calibration plot: visualizing agreement between model-predicted (*Ped-CMU ExFPS*) and observed proportion of extubation failure.

Abbreviations: *Ped-CMU ExFPS*: Pediatric CMU Extubation Failure Predictive Score.

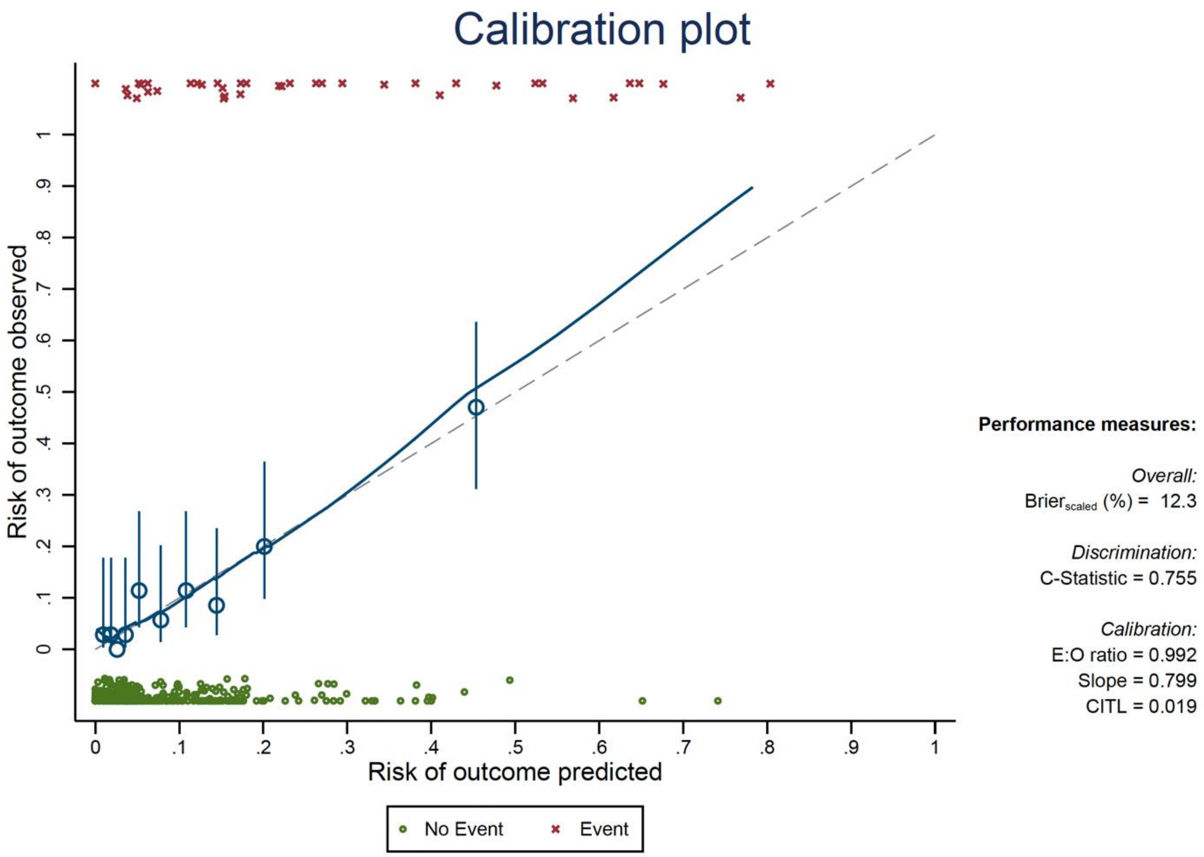

Supplementary Figure 2. Internal validation with bootstrapping procedure with 500 replicates showed an apparent AUC of 0.80 (range 0.72 to 0.88) and a test AUC of 0.76 (range 0.68 to 0.83). The optimism of AUC was 0.05 (range 0.04 to 0.05).

Abbreviations: AUC: the receiver operating characteristic.

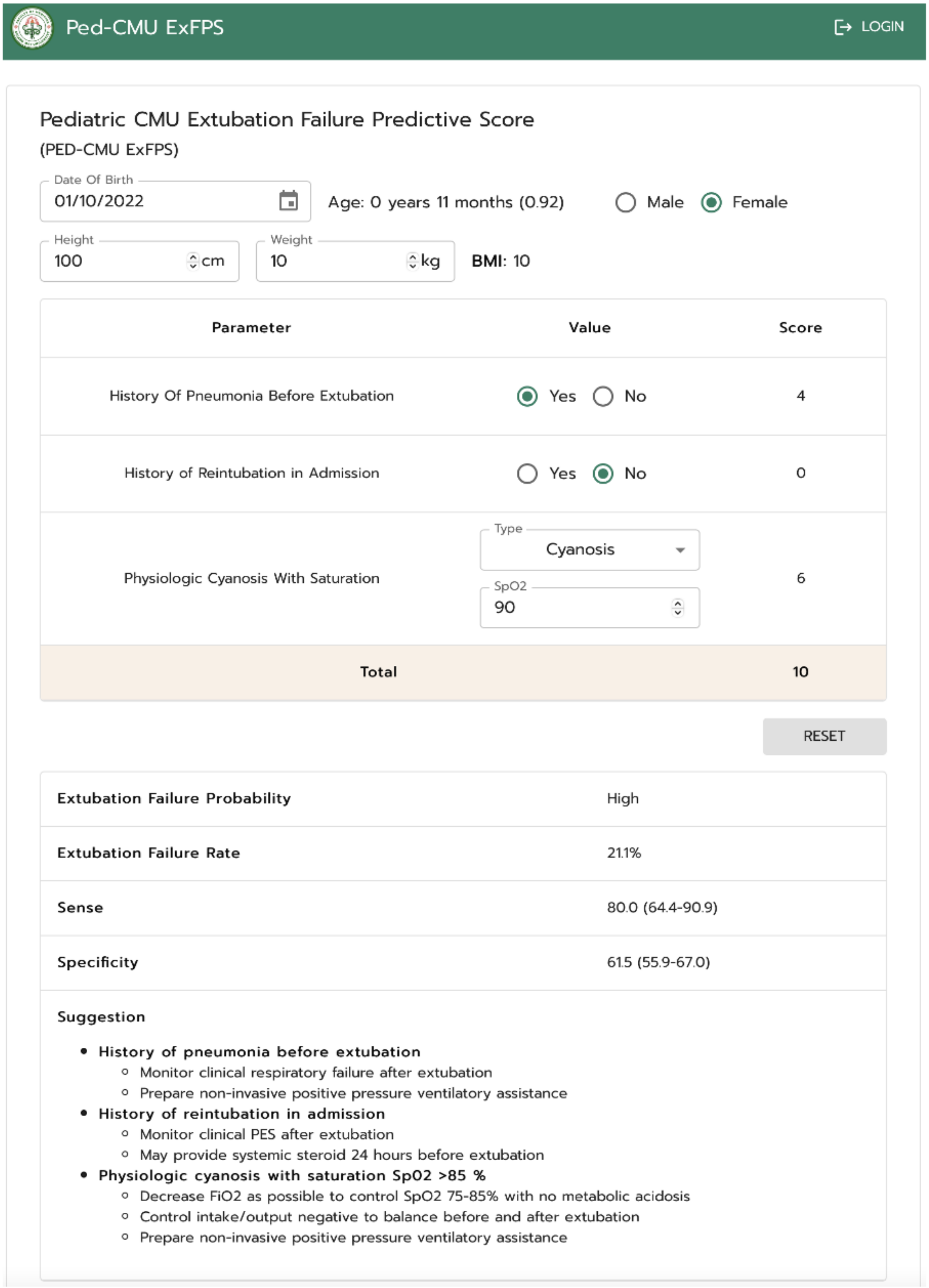

Supplementary Figure 3. An interface of the *Ped-CMU ExFPS*.

Abbreviations: *Ped-CMU ExFPS*: Pediatric CMU Extubation Failure Predictive Score.

Pediatric CMU Extubation failure predictive score (Ped-CMU ExFPS)

Parameter	Value	Score
**History of pneumonia before extubation**	Yes	4
	No	0
**History of reintubation in admission**	Yes	10
	No	0
**Physiologic cyanosis with saturation**	Cyanosis (SpO2 ≤85)	0
	Acyanosis	1
	Cyanosis (SpO2 >85)	6
**Total (maximum 20)**		

Supplementary Figure 4. Thee *Ped-CMU ExFPS: A Pen and Paper Version*

Abbreviations: *Ped-CMU ExFPS*: Pediatric CMU Extubation Failure Predictive Score.

1.2 Supplementary Tables

Supplementary Table 1. Potential predictors of extubation failure based in an univariable logistic regression analysis.

Variable	Odd	95% CI		P-value	ROC	95% CI	
		Lower	upper			Lower	upper
Laboratory values within 36 hr before extubation							
**Venous blood gas**							
pH	13.11	0.02	5817.93	0.408	0.56	0.45	0.68
pCO2, mmHg	0.98	0.94	1.03	0.410	0.54	0.42	0.67
Bicarbonate, mmol/L	0.99	0.92	1.06	0.796	0.51	0.40	0.63
Venous oxygen saturation (SvO2)	1.00	0.98	1.01	0.994	0.50	0.38	0.62
Lactate, mmol/L	1.08	0.83	1.43	0.540	0.68	0.53	0.84
Creatinine, mg/dL	0.79	0.24	2.55	0.690	0.51	0.40	0.61
Sodium, mEq/L	1.01	0.94	1.09	0.710	0.50	0.40	0.61
Potassium, mEq/L	1.43	0.87	2.34	0.155	0.57	0.47	0.67
Phosphorus, mg/dL	1.02	0.99	1.05	0.132	0.53	0.44	0.62
Magnesium, mg/dL	1.11	0.96	1.28	0.141	0.54	0.45	0.64
Hemoglobin, g/dL	0.99	0.86	1.15	0.942	0.51	0.40	0.62
Hematocrit %	1.00	0.96	1.05	0.846	0.51	0.40	0.62
White blood cell, count 10^9/L	1.00	0.99	1.00	0.070	0.58	0.48	0.67
Platelets, count 10^9/L	1.00	0.99	1.00	0.881	0.50	0.40	0.61
AST, U/L	1.00	0.99	1.00	0.582	0.54	0.41	0.67
ALT, U/L	1.00	0.99	1.00	0.809	0.50	0.39	0.62
Total bilirubin, mg/dL	0.94	0.72	1.23	0.633	0.53	0.41	0.65
Albumin, g/dL	1.06	0.54	2.05	0.874	0.53	0.40	0.66

Abbreviations: AST: aspartate transaminase; ALT: alanine aminotransferase.

Supplementary Table 2. Overall probability of extubation failure after incorporating PES into the *Ped-CMU ExFPS.*

**Ped-CMU ExFPS**	**Baseline failure probability (%)**	**Failure probability incorporated with PES (%)**	
		**PES positive**	**PES negative**
0	3.60	7.60	2.19
1	4.49	9.39	2.75
4	8.60	17.16	5.35
5	10.60	20.68	6.54
6	12.99	24.73	8.22
10	27.32	45.27	18.40
11	32.14	51.02	22.21
14	48.62	67.55	36.21
15	54.38	72.39	41.70
16	60.00	76.76	47.39
20	79.08	89.26	69.40

Abbreviations: *Ped-CMU ExFPS*: Pediatric CMU Extubation Failure Predictive Score; PES: post extubation stridor.
